# Wanderings of Pus

**Published:** 1866-03

**Authors:** Leon F. Harvey


### WANDERINGS OF PUS.

BY LEON F. HARVEY, M. D.

Pus, that product of inflammation, which the medical man so often desires to see, and uses means to produce, knowing thereby speedy relief and certain cure is sure to follow its production and expulsion; yet, which at other times, he is so anxious to prevent, feeling that when suppuration intervenes, the death of the part or the patient may often ensue, frequently makes curious journeys about the human system. Its tendency for burrowing is very great, making slow but sure progress through a tissue, as water through a small crevice in the earth, with its noiseless but onward movement; it breaks away all barriers, and at last comes to the surface. We are many times puzzled to find the cause of pus exuding from a fistulous opening. No sign of disease about it; no complaint of pain or soreness on touch, but a knowledge that pus often makes its exit, inches and even feet, from the seat of the disease, causes us to look far for the source of the never failing spring. We shall endeavor to show, by attention to marked cases that have come under our own observation, and of others, the necessity of examining the condition of the teeth and the adjacent parts, when fistulous openings are observed near to, or even remote as the clavicle is, from the mouth. Many months of tedious treatment may be averted by examining the teeth and jaws when such a case is brought to our notice. There is a rule which we too often neglect in the treatment of disease, namely, that of cause and effect. We know that disease cannot exist without a cause, but ignoring its presence, the effect claims all our attention. And thus it has been with scores of fistulous openings; failures to find, or even to look, for the cause so often found in a carious tooth, a diseased alveolus or even a small portion of a root. A great deal of this arises from the unwillingness of the physician to recognize the important action which the teeth and their remains have upon the system. They only look upon them as so many grinders, placed in a position to divide the food, as the mill is used to grind the grain, but that duty performed, they exert no influence till again brought into requisition. To be sure they will not dispute the fact that a tooth will ache; yet will laugh at the idea that many of the functions may be deranged by it. The intimate relation between the teeth and the organism can not be too closely studied. The first case which we shall relate as showing the wanderings of pus is as follows: Mr. A. R.——, æt. 30, of a nervous temperament, whose habits were good, consulted us in regard to a swelling on the apex of his chin, from which there was an almost constant dripping of pus upon his shirt bosom, to his great annoyance. He had been troubled in this manner for about three years, but had been unable to find the cause or obtain relief. With a very small flexible silver probe, an exploration was made, and striking the central incisor, supposed that to be the seat of the trouble; but on further exploration, became convinced that the second right bicuspid of the lower jaw gave rise to the discharge. It was perfectly sound and but slightly discolored; but on making an opening through the alveolus to the root, found that it was dead. By keeping that aperture open, the pus passed through it, and, in a few weeks, there was no discharge from the chin. The second is the most marked case, for the pus had traveled a much greater distance.

Miss C. L.——, æt. 25, of a nervous temperament, came to ask our advice about a swelling that had existed for two or three years, upon the left clavicle. It was about the size of a child's fist, and would discharge of itself or upon being opened, a gill of pus. She had consulted many doctors, here and in Canada, mostly quacks, we presume, but they all could give her no aid. Whilst talking with her about the affection, there was noticed a stain upon the neck following the course of the muscles to the swelling, and thinking that the teeth might give rise to the trouble, made an examination of them. Found that the first molar of the left side, lower jaw, had been removed, but the gum over its sockets was red, and sore upon pressure. Believing that roots are never entirely covered with integument, did not think it possible that any could be present here; but on probing, found there were, and, on removing them, the healing of the parts proves that they were the cause of the swelling. In this case we clearly see the importance of a careful examination of the teeth and their sockets. The discharge of pus was very great, and, had not the source of it been found, the patient could not much longer have endured the drain.

Case No. 3 is related at page 326, vol. 6 of the American Journal of Dental Science, Old Series. A girl, æt. 16, was brought to M. Geessant with a fistulous opening in the anterior region of the neck, the consequence of an abscess. On examing the teeth, he found that one of the inferior canine teeth was discolored, and that he could introduce a probe between the tooth and the alveolus: the tooth was removed and the fistula cured without delay.

Case No. 4 may be found at page 184, vol. 8, of the Dental News Letter, related by Jno. J. Watt, Edinburg, Scotland.

A young girl, about 10 years of age, was brought to me in the month of November last, with a fistulous opening of the left cheek, opposite the first permanent molar, from which pus had been discharged several months. On examining the mouth, I found the first left inferior molar with the crown completely decayed, which I extracted, and in the course of two or three weeks the fistulous opening was entirely healed up. A few weeks ago, the same girl was brought back to me, to ask my opinion regarding a discharge of pus from the right ear. On looking into the mouth, I found the first permanent inferior molar of that side very much in the same state of decay as the one on the opposite side, which I had previously extracted; and as I had little doubt of the discharge being caused by the irritation produced by the carious tooth, it was also extracted, and with complete success, as it ceased in two or three days, and there has been no return since.

Case No. 5, the last that I shall cite, is recorded in vol. 11, page 148, of the Dental News Letter.

R. S.——, by occupation a coachman, footman, &c., to an old gentleman; called upon me two years ago to get something done for a sore on the centre of his left cheek. He said it had been a boil which suppurated and broke about two months previously. His face was much swollen, and, as he had to wait at his master's table, it rendered him unfit for his work. The patient had consulted another surgeon, and had tried various remedies, but could not get it healed. He was otherwise the picture of health. I thought it might be some chronic affection of the parotid gland, strumous or otherwise. I gave him some zinc lotion to inject into the opening, and to apply a bit of rag, dipped into the same, and covered with oil silk. In about three weeks he came to tell me that the sore was healed, but his cheek was swollen. I gave him iodine to apply over it. There was an ugly cicatrix where the sore had been. In the course of another month the patient came again, presenting an abscess ready to burst in the old place. I opened it, told him to poultice it a few days, and then use the former treatment. In about three months he called again, and told me that after he last saw me, being useless in his situation, he went home to Edinburgh, where he saw Professor Syme, who gave him something to use, and that the sore did not heal for six weeks after I opened it. The professor told him that the sore arose from a wisdom tooth coming up, for which there was not room in the jaw, and advised him, if the sore did not heal, or should trouble him again, to have the adjacent tooth extracted. His face was now much swollen, and an abscess was evidently forming again. I examined his mouth, and saw that he had got the upper wisdom tooth only, and that there was evidently a want of space for that below. I accordingly extracted the second molar tooth, after which the swelling gradually subsided, and the wisdom tooth filled up the vacant space. He was now permanently cured. We give this last case as an evidence that not only disease, but pressure may afford much trouble, and that we are not to cease observations of the mouth if no decayed or dead teeth are found; but the wisdom teeth, in regard to which we are consulted so often, should not be overlooked in our searchings for the cause of a flow of pus. We could multiply cases, but deem those we have given sufficient to impress upon the minds of all the importance of a thorough examination of everything connected with the mouth, in looking for the fountain head of pus.

